# Novel Mutations in Obesity-related Genes in Turkish Children with Non-syndromic Early Onset Severe Obesity: A Multicentre Study

**DOI:** 10.4274/jcrpe.galenos.2019.2019.0021

**Published:** 2019-11-22

**Authors:** Ayşehan Akıncı, Doğa Türkkahraman, İbrahim Tekedereli, Leyla Özer, Bahri Evren, İbrahim Şahin, Tarkan Kalkan, Yusuf Çürek, Emine Çamtosun, Esra Döğer, Aysun Bideci, Ayla Güven, Erdal Eren, Özlem Sangün, Atilla Çayır, Pelin Bilir, Ayça Törel Ergür, Oya Ercan

**Affiliations:** 1 nönü University Faculty of Medicine, Department of Pediatric Endocrinology and Diabetes, Malatya, Turkey; 2 Antalya Training and Research Hospital, Clinic of Pediatric Endocrinology and Diabetes, Antalya, Turkey; 3 İnönü University Faculty of Medicine, Department of Molecular Genetics, Malatya, Turkey; 4 Yüksek İhtisas University Faculty of Medicine, Department of Molecular Genetics, Ankara, Turkey; 5 İnönü University Faculty of Medicine, Department of Endocrinology and Diabetes, Malatya, Turkey; 6 Antalya Training and Research Hospital, Clinic of Molecular Genetics, Antalya, Turkey; 7 Gazi University Faculty of Medicine, Department of Pediatric Endocrinology and Diabetes, Ankara, Turkey; 8 Göztepe Training and Research Hospital, Clinic of Pediatric Endocrinology and Diabetes, İstanbul, Turkey; 9 Uludağ University Faculty of Medicine, Department of Pediatric Endocrinology and Diabetes, Bursa, Turkey; 10 Başkent University Faculty of Medicine, Department of Pediatric Endocrinology and Diabetes, Adana, Turkey; 11 Erzurum Training and Reseach Hospital, Clinic of Pediatric Endocrinology and Diabetes, Erzurum, Turkey; 12 Ankara University Faculty of Medicine, Department of Pediatric Endocrinology and Diabetes, Ankara, Turkey; 13 Ufuk University Faculty of Medicine, Department of Pediatric Endocrinology and Diabetes, Ankara, Turkey; 14 İstanbul University-Cerrahpaşa, Cerrahpaşa Faculty of Medicine, Departments of Pediatric Endocrinology and Diabetes, and Adolescent, İstanbul, Turkey

**Keywords:** Early, onset, severe obesity, novel mutations

## Abstract

**Objective:**

Non syndromic monogenic obesity is a rare cause of early onset severe obesity in the childhood period. This form may not be distinguishable from other forms of severe obesity without genetic analysis, particularly if patients do not exibit any physical abnormalities or developmental delay. The aim of this study was to screen 41 different obesity-related genes in children with non-syndromic early onset severe obesity.

**Methods:**

Children with severe (body mass index-standard deviation score >3) and early onset (<7 years) obesity were screened by next-generation sequencing based, targeted DNA custom panel for 41 known-obesity-related genes and the results were confirmed by Sanger technique.

**Results:**

Six novel variants were identified in five candidate genes in seven out of 105 children with severe obesity; two in SIM1 (p.W306C and p.Q36X), one in *POMC* (p.Y160H), one in *PCSK1* (p.W130G fs Ter8), two in *MC4R* (p.D126E) and one in *LEPR* (p.Q4H). Additionally, two previously known variations in *MC4R* were identified in four patients (p.R165W in three, and p.V166I in one).

**Conclusion:**

We identified six novel and four previously described variants in six obesity-related genes in 11 out of 105 childrens with early onset severe obesity. The prevalence of monogenic obesity was 10.4% in our cohort.

**What is already known on this topic?**

Non-syndromic, early onset, severe obesity is usually a result of mutations in a single gene (monogenic), such as *SIM1, POMC, PCSK1, MC4R, LEP* and *LEPR*, that directly influence the leptin-melanocortin pathway which regulates satiety.

**What this study adds?**

We identified six different novel variants within five obesity-related genes *(SIM1, POMC, PCSK1, MC4R* and *LEPR)* in seven of 105 childrens with early onset severe obesity in a Turkish population.

## Introduction

Common forms of obesity are caused by a combination of environmental and behavioral factors, together with an underlying genetic predisposition to obesity. The etiology of childhood obesity is multifactorial. Non syndromic early-onset severe obesity is usually monogenic, while other forms of obesity are polygenic and occur due the cumulative effect of multiple susceptibility genes which regulate energy intake and expenditure. It has been reported that non-syndromic monogenic obesity is very rare, not exceeding 7% of childhood obesity cases (1,2,3). However, this ratio varies with ethnic characteristics and the proportion of consanguineous couples within any given population. To date mutations in several genes which cause the development of early-onset, severe obesity in children have been described. However, with advances in genetic testing, more genetic causes of obesity continue to be identified. Most of these genes, such as *LEP, LEPR, SIM1, POMC, PCSK1 *and* MC4R*, are involved in the central regulation of satiety via the leptin-melanocortin signaling pathway. Therefore variants in any of these genes cause overt changes in food intake, body weight and energy expenditure and are also associated with some forms of neuroendocrine and immune dysfunction (4,5,6).

Syndromic obesity is usually diagnosed clinically with features such as hyperphagia, early-onset severe obesity, developmental delay or other findings caused by defects in the responsible gene. However, in some types of monogenic obesity, it may not be possible to diagnose the underlying genetic defect solely on the basis of clinical findings. For example, mutations in *MC4R* lead to the most prevelant form of monogenic obesity and, because the clinical features resemble those found in exogenous obesity, differential diagnosis can only be confirmed by detection of genetic variants (4,5,6,7). With the exception of leptin deficiency due to leptin gene mutations, treatment options are limited in early-onset severe obesity. Newly available, targetted drugs will offer a novel therapeutic option for those patients with monogenic obesity due to *MC4R* or *POMC* dysfunction (8,9). Consequently, genetic testing should be advocated in children with early onset severe obesity as they may be suitable candidates for current or promising new drugs such as MC4R agonists. The present study, has been conducted to assess the variants of 41 different obesity-related genes in Turkish children with non-syndromic early onset severe obesity.

## Methods

The study population was selected from among severe obese patients referred to our center at İnönü University, Malatya, Turkey, for genetic analysis from different centers in geographically diverse parts of Turkey. Inclusion criteria for children and adolescents were obesity onset at less than seven years of age and a body mass index-standard deviation score (BMI-SDS) >3. Patients taking any drugs or followed up with any specific endocrine disorders, such as Cushing syndrome or hypothyroidism, and those with syndromic features were not included in the study. The study protocol was approved by the regional Ethical Committees (Malatya Clinical Research Ethics Committee, 21.01.2018, no: 2018-20), and informed consent was obtained from the parents of all children before their participation.

### Anthropometric Measurements

All patients were examined in the morning after an overnight fast. Height and weight were measured by experienced nurses from the pediatric endocrinology outpatient clinic. BMI was calculated as body weight in kilograms divided by the square of the height in meters. BMI and BMI-SDS were calculated using age and gender specific percentiles of Turkish children from established reference data (10).

### DNA Preparation

Genomic DNA was isolated from peripheral blood mononuclear cells using the QiAamp DNA Blood Mini Kit (cat. no. 51106, Qiagen, Hilden, Germany). DNA purity and quality was confirmed by agarose gel electrophoresis. DNA concentration was measured by Qubit (Life Technologies, Singapore). Before the library preparation, appropriate dilution was made for each sample.

### Next Generation Sequencing

Sequencing libraries were prepared according to the manufacturer’s instructions using CDHS-1346Z-901 QIASeq^™^ Targeted DNA Custom Panel (ref. no. 333525, Qiagen, Hilden, Germany) that includes all exomes with 10bp exon-intron junctions of 41 target genes (*DYRK1B, LEP, LEPR, MC4R, NR0B2, POMC, UCP3, ADRB2, ADRB3, AGRP, MC3R, NTRK2, PCSK1, SIM1, CARTPT, ENPP1, PPARG, PPARGC1B, PYY, SDC3, UCP1, ADIPOQ, NAMPT, CFD, RETN, PPARGC1A, CCK, NPY, SLC2A4, ADD1, SREBF1, PTPN1, IRS-1, GHRL, BDNF, NEGR1, SH2B1, GIPR, TMEM18, FTO, SLC22A1*). Briefly, the samples were enzymatically fragmented and molecularly barcoded and passed through the stages of library generation, target enrichment, sample indexing and amplification. The concentration of each library was determined by using Qiaseq Library Quant Assay Kit (ref. no. 333314, Qiagen, Hilden, Germany) according to the manufacturer’s instructions. Each library was diluted to 4 nM, and pooled in equimolar ratio. The final pool was denatured with freshly prepared 0.2 N NaOH and then diluted to 20 pM and sequenced as 251x2 bp paired-end chemistry according to the sequencer manufacturer’s instructions (MiSeq, Illumina, San Diego, CA) (11).

### Sequencing Data Analysis

Demultiplexed FASTQ files were processed individually using Qiagen Bioinformatics solutions. Secondary analysis was performed by using Qiagen, QCI Analyze Universal 1.5.0. Tertiary analysis and interpretation were performed using Qiagen Clinical Insight Interpret (all programs from Quiagen, Hilden, Germany).

### Sanger Sequencing

Detected variants were also analysed and confirmed by Sanger sequencing according to the manufacturer’s protocols. The amplicons were analyzed by direct sequencing with ABI 3500 (Life Technologies, Waltham, Massachusetts, USA). Analysis of sequence results was performed by Mutation Surveyor Programme (SoftGenetics, USA).

### Data Analysis

Mutations and/or polymorphisms were screened for using next-generation sequencing. All the genes that were investigated have various roles in energy homeostasis, such as energy intake, energy expenditure, adipose tissue functions and glucose metabolism. Genetic variant pathogenicity was examined using the following standard *in silico* analyses; MutationTaster, PolyPhen-2, CADD, Stratum and I-Mutation-2.0: prediction. Novel mutations detected were verified by Sanger sequencing (12,13,14).

## Results

**Figure 1:**
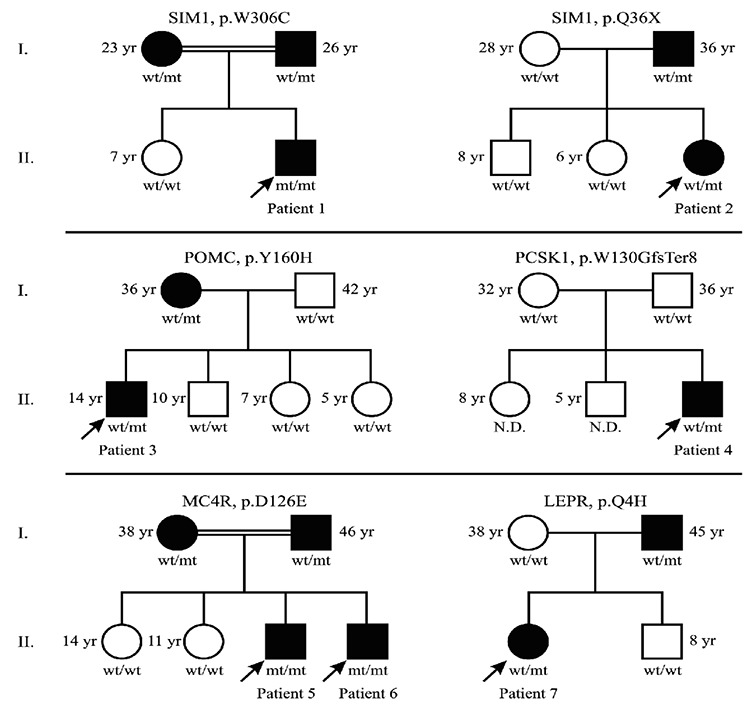
Pedigrees of the families bearing novel variants in obesity related genes. Arrows indicate probands in each family. Genotypes were defined as wild type (wt) or mutant (mt) for corresponding variations

A total of 105 patients meeting the inclusion criteria were included in the study. Table 1 shows the key clinical and genetic characteristics of the children carrying the obesity-related gene variations. We described six novel mutations in five candidate genes in seven out of the 105 patients, and previously described mutations in *MC4R* detected in four patients. The novel variations detected were two in *SIM1*, one in *POMC*, one in *PCSK*, one in *LEPR* and two in *MC4R* (Table 1). Family members of these affected children were also genetically screened for the same pathogenic variants. Family pedigrees of the children carrying the novel variations are shown in Figure 1.

### Genetic Results

Table 1 shows the key clinical characteristics of the patients carrying novel mutations. We identified six novel mutations potentially contributing to the severe obesity in these subjects. In Patient 1, a novel homozygous *SIM1* variant (p.W306C, c.918 G>T in exon 8) was detected. He was two years and three months of age at the time of the study, his BMI-SDS was 5.6, and obesity onset age was one and a half years. His birth weight was 2900 g. He had no other endocrinological or developmental abnormalities. His parents were second degree relatives. His father and mother were also obese and both were heterozygous for the same variation.

The second patient (Patient 2) carrying a novel heterozygous *SIM1* variant (p.Q36X, c.106 G>T in exon 1), was five years old, her birth weight was 2300 g, her BMI-SDS was 4.7 and she had severe obesity from two years of age. Her growth velocity and developmental history were normal and she had no additional endocrinological or developmental abnormalities. There was no consanguinity in her family. Her father was obese and heterozygous for the same variants.

A novel heterozygous *POMC* variant (p.Y160H, c.478 T>C in exon 3) was detected in a male patient who was 14 years old (Patient 3). He had no other abnormalities except severe obesity and hyperphagia. There was no consanguinity in his family, but his obese mother was heterozygous for the same variation.

In Patient 4, a novel heterozygous *PCSK1* variant (p.W130G fs Ter8, c.388delT) was detected. He was two years and four months old and had no endocrinological abnormalities. His parents were not obese and had no genetic variation detected on our panel.

Two siblings in the same family (Patients 5 and 6) were homozygous for a novel *MC4R* variant (p.D126E, c.378C>A in exon 1). They had severe obesity, intractable hyperphagia and accelerated growth which is typical for *MC4R* deficiency. Their parents were severely obese and close relatives, and they were both heterozygous for the same variantion.

**Figure 2:**
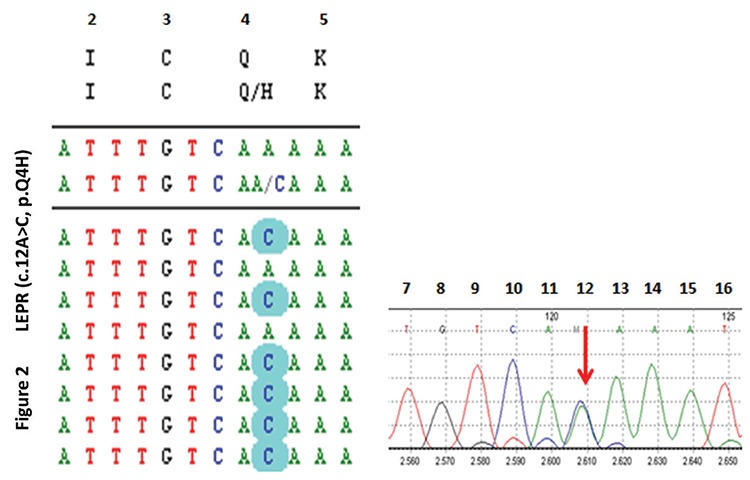
DNA sequencing by the next-generation sequencing (NGS) method revealed a novel heterozygous c.12A>C, p.Q4H mutation in *LEPR*. Related mutations are highlighted in NGS sequences and designated by red arrows in Sanger sequences

The last female patient (Patient 7) was 14 years old, severely obese and heterozygous for a novel *LEPR* variant (c.12A>C, p.Q4H in exon 3) (Figure 2). Her obese father was heterozygous for the same variant.

In addition to these novel variants, previously described mutations in *MC4R* were found in four patients (p.R165W, c.493C>T in exon 1 in three of four, and p.V166I, c.496G>A in exon 1 in one) (15,16).

## Discussion

In this study, variants in 41 genes which are known to be involved in causing obesity in patients with non-syndromic early onset severe obesity were investigated. Two novel *SIM1* variants in two unrelated patients, a novel *POMC* variant, a novel *PCSK 1* variant, two siblings with the same *MC4R* variant and a novel *LEPR* variant were identified in our cohort.

Single-minded-1 gene *(SIM1*) is located on chromosome 6q16.3-q21 and consists of 11 exons spanning 75kb. *SIM1* encodes a hypothalamic transcription factor in the basic helix loop helix/Per Arnt Sim (bHLH-PAS) family. Its main function has been described as the formation of the paraventricular nucleus of the hypothalamus which is critical for food intake regulation. *SIM1* also plays an important role in the regulation of energy homeostasis by interacting with the melanocortin signalling pathway and loss-of-function variants in this gene are one of the few known causes of monogenic obesity in both humans and mice (17,18). Recently, it has been reported that chromosomal abnormalities such as translocation between chromosome 1p22.1 and 6q16.2, deletion of the 6q16.2 region and heterozygous point mutations in the *SIM1* region are responsible for early-onset severe obesity in humans (19,20,21). In these reports, patients had increased fat mass with increased body fat percentage in addition to hyperphagia, increased linear growth, learning disabilities and Prader-Willi-like phenotype. Experimentally, it has been observed that homozygous *Sim1* knockout mice *(Sim1* -/-) do not survive due to lack of the hypothalamic neurons which produce multiple neuropeptides including oxytocin, vasopressin, corticotropin-releasing hormone, thyrotropin-releasing hormone and somatostatin. However, heterozygous mice (*Sim1 +/-)* develop partial failure of hypothalamic neurons resulting in hyperphagia and obesity similar to *mc4r-mutant mice *(22). In our study group, we described one patient with a homozygous missense *SIM1* variant (p.W306C, c.918 G>T in exon 8) and another patient with heterozygous nonsense *SIM1* variant (p.Q36X, c.106 G>T in exon 1). The homozygous patient had severe obesity due to hyperphagia from eighteen months of age and his obese parents were also heterozygous for the same *SIM1* variant. This p.W306C variant is located in the PAS domain, which has a critical role in *SIM1* activity (23). Stratum and I-Mutant 2.0 prediction analysis suggest that the Gibbs free energy (delta delta G, DDG) value of this mutant protein would be -1.7 and CADD score was 35, indicating a decrease in the stability of the mutant protein structure. Therefore, this variant is likely to be pathogenic because of changes in the protein structure and redox status leading to reduced SIM1 activity. Previously, pathogenic variants have been described in this region (23,24,25), and it appears that this new variant located in the same region is also pathogenic. In addition, and contrary to what might be expected, identification of accelerated growth at his physical examination and the resemblance of his phenotype to the *MC4R* variants led us to hypothesize that this *SIM1* variant might induce considerable functional loss in MC4R activation. Needless to say, functional studies would be required to confirm this.

The mother of Patient 2, in whom a novel heterozygous nonsense *SIM1* variants (p.Q36X, c.106 G>T in exon 1, CADD score: 37) was identified, did not carry the same variant, whereas his obese father was haploinsufficient for p.Q36X. This new variant located in the bHLH domain of *SIM1* is predicted to play a significant role in DNA dimerization and binding, so it is likely to be pathogenic according to Polyphen-2 and CADD analysis. In addition to the critical location of this variant, its pathogenity is enhanced because it also produces a premature stop codon resulting in a truncated protein. Previously, a loss-of-function, heterozygous *SIM1* variant (T46R) was described in the same region (24,25). Although most of the heterozygous *SIM1* variants that cause obesity have been described as causing growth retardation and a Prader-Willi-like syndrome in addition to the accompanying obesity (24,25), developmental and intellectual capacity was normal in our patient.

Proopiomelanocortin (POMC) is produced by the POMC/CART (cocaine and amphetamine-related transcript) neurons in the hypothalamus, and is the precursor of adrenocorticotropic hormone (ACTH), beta-endorphin, beta-lipotropin (beta-LPH), corticotropin-like intermediate peptide (CLIP) and α-, β- and γ-melanocyte-stimulating hormones (MSH), some of which regulate melanin synthesis, adrenal functions and inhibit food intake through interaction with the MC4R signalling pathway (26,27,28). Homozygous loss-of-function mutations in *POMC* have been reported to be very rare and a cause of severe obesity, ACTH deficiency and hypopigmentation in mice and humans (29,30). It is suggested that the MC4R signalling pathway is affected secondary to the impairment of interaction with MC4R and α-MSH in heterozygous missense *POMC* variants without complete POMC deficiency, and subsequently severe obesity develops in humans (29,30,31). In this study, a novel heterozygous *POMC* variant (p.Y160H) was described in a patient with early-onset, severe obesity whose obese mother was also affected by the same variant. This variant was located in the CLIP region of the ACTH domain of *POMC*. The DDG value of this mutant protein was -1.62 kj/mol, predicted by Stratum and I-mutant 2.0 analysis, and CADD score was 25.8 leading to a decrease in the stability of the mutant protein. PolyPhen-2 analysis predicted that this novel variant is likely to be pathogenic. Although the function of CLIP is not fully understood in humans, it is considered that variants affecting this region may confer the phenotype through an altered MC4R signalling pathway.

The proprotein convertase subtilisin/kexin type 1 gene *(PCSK1*) encodes the prohormone convertase enzyme (PC1/3) and is abundantly expressed in the hypothalamus (32). PC1/3 deficiency is described as an autosomal recessive disorder. Although heterozygous PC1/3 deficiency is associated with obesity, homozygote loss-of-function mutations usually present with early onset severe obesity and hyperphagia in addition to malabsorptive diarrhea in the neonatal period, central diabetes insipidus, reactive hypoglycemia and hypoadrenalism (33,34,35).

However, the described phenotype may be variable depending on which parts of the *PCSK1* gene structure have been affected. In Patient 4, a novel heterozygous frameshift *PCSK1* variant (p.W130G fsTer8, C388delT) was found. However, the same variant was not present in his parents. This novel variant is located in a catalytic domain of *PCSK1* and leads to a frameshift mutation and deletion followed by stop-codon that is predicted to produce a non-functional truncated protein. Its CADD score was 36. It has been described that pathogenic variants within the same domain reduce the PCSK1 activity (34,35). Therefore, it seems highly likely that this novel variant would be pathogenic.

MC4R is the receptor for α-MSH and plays a key role in controlling energy homeostasis, food intake and satiety. *MC4R* mutations are the most common genetic cause of monogenic obesity and also contribute in polygenic forms. Loss-of-function *MC4R* mutations are associated with early-onset severe obesity due to hyperphagia, hyperinsulinemia and increased linear growth. Currently more than 150 variants have been identified, and the prevalence of pathogenic *MC4R* variants reported in various obese populations is highly variable, ranging from 0.5% to 6% (1,36,37,38). We found a novel homozygous *MC4R* variant, D126E, in exon 1 in two siblings. This mutation is located on the helical transmembrane domain/putative ligand binding site (NCBI-search tool). Its DDG value was -1.33 kj/mol predicted by Stratus and I-Mutation 2 prediction, and CADD score was 24.5, suggesting a possible decrease in the function of the mutant protein. This variant may lead to a decrease in the binding capacity of MC4R to α-MSH, as previously described in the pathogenic variants, I137T, R165W and G98R located in the same region of *MC4R* (39,40,41,42). Thus this novel variant, D126E, is likely to be pathogenic. Our affected siblings were extremely obese, and they had increased height velocity for age. Their parents were heterozygous for the same variant and they were also severely obese. Additionally, we found two different previously described mutations in *MC4R* in four patients (p.R165W, c.493C>T in exon 1 in three, and p.V166I, c.496G>A in exon 1 in one) (Table 1)*.*

Leptin and *LEPR* mutations are associated with early onset severe obesity, severe hyperphagia and some neuroendocrine abnormalities, such as hypogonadotropic hypogonadism, impaired growth hormone secretion and hypothalamic hypothyroidism (43,44). Patient 7 had a novel heterozygous *LEPR* mutation (p.Q4H, c.12A>C in exon 3, Figure 2). She was severely obese and had no endocrinopathy. The heterozygous *LEPR* variant detected in this patient is located in the signal peptide and may destroy protein synthesis and/or processing (sorting and location). Its DDG was -1.13 kj/mol, signifying a decrease in protein stability and CADD score was 10. Deletions causing dysfunction in the signal peptide located in the extracellular domain of *LEPR* have been reported (44). Although it is hard to speculate about this variant without performing an analysis to confirm abnormal protein processing, the patient’s phenotype and heterozygosity of the obese father for the same variant led us to suppose that this novel variant is most likely pathogenic. However, definitive functional analysis should be performed to confirm pathogenicity.

In the literature, there are a few similar studies detecting obesity-related genes with a targeted DNA custom panel. In a recent report by Foucan et al (45), 59 obesity-related genes were screened by next-generation sequencing in 25 obese children in Guadeloupe Island and five mutations in *MC4R, SIM1, SH2B1 *and *NTRK2* genes were described. The prevalence of monogenic obesity in this cohort was 10% which is similar to the present study.

### Study Limitations

There were some limitations associated with our study. We could not conduct a functional analysis of the mutant genes. However, we believe that the relationship between the genotype and phenotype of the patients and the assessment of possible functional losses that would result from the novel mutations provide compelling evidence of the effect of these novel mutations.

## Conclusion

We identified six different novel variants within five obesity-related genes (*SIM1, POMC, PCSK1, MC4R* and *LEPR*) in seven out of 105 children with early-onset severe obesity in a Turkish population. Additionally, we found previously known mutations in the *MC4R* gene in four patients, thus monogenic obesity prevalence was 10.4% in our cohort. In order to understand whether these novel variants are specific to the Turkish community in which consanguineous marriages are common, further broad-based genetic screening studies are needed.

## Figures and Tables

**Table 1:**
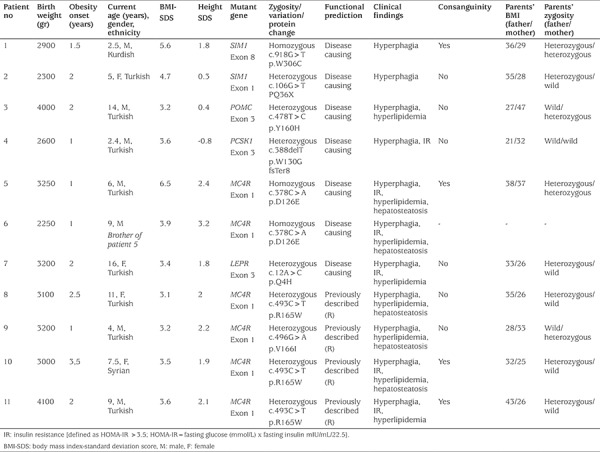
Clinical and genetic characteristics of the patients with obesity-related gene variations

## References

[1] Farooqi IS (2005). Genetic and hereditary aspects of childhood obesity. Best Pract Res Clin Endocrinol Metab.

[2] Huvenne H, Dubern B, Clement K, Poitou C (2016). Rare genetic forms of obesity: Clinical approach and Current treatments in 2016. Obes Facts.

[3] Dayton K, Miller J (2018). Finding treatable genetic obesity: stategies for success. Curr Opin Pediatr.

[4] da Fonseca ACP, Mastronardi C, Johar A, Arcos-Burgos M, Paz-Filho G (2017). Genetics of non-syndromic childhood obesity and the use of high-throughput DNA sequencing technologies. J Diabetes Complications.

[5] Singh RK, Kumar P, Mahalingam K (2016). Molecular genetics of human obesity: A comprehensive review. C R Biol.

[6] Mason K, Page L, Balıkcioglu PG (2014). Screening for hormonal,monogenic, and syndromic disorders in obese infants and children. Pediatr Ann.

[7] Lubrano-Berthelier C, Dubern B, Lacorte JM, Picard F, Shapiro A, Zhang S, Bertrais S, Hercberg S, Basdevant A, Clement K, Vaisse C (2006). Melanocortin 4 receptor mutations in a large cohort of severely obese adults: Prevalence, functional classification,genotype-phenotype relationship,and lack of association with binge eating. J Clin Endocrinol Metab.

[8] Farooqi IS, Jebb SA, Langmack G, Lawrence E, Cheetham CH, Prentice AM, Hughes IA, McCamish MA, O’Rahilly S (1999). Effects of recombinant leptin therapy in a child with congenital leptin deficiency. N Engl J Med.

[9] Kühnen P, Clément K, Wiegand S, Blankenstein O, Gottesdiener K, Martini LL, Mai K, Blume-Peytavi U, Grüters A, Krude H (2016). Proopiomelanocortin deficiency treated with a melanocortin-4-receptor agonist. N Engl J Med.

[10] Neyzi O, Bundak R, Gökçay G, Günöz H, Furman A, Darendeliler F, Baş F (2015). Reference values for weight, height, head circumsference and body mass index in Turkish children. J Clin Res Pediatr Endocrinol.

[11] Shendure J, Ji H (2008). Next-generation DNA sequencing. Nat Biotechnol.

[12] Quan L, Lv Q, Zhang Y (2016). STRATUM: Structure-based stability change prediction upon single-point mutation. Bioinformatics.

[13] Adzhubei IA, Schmidt S, Peshkin L, Ramensky VE, Gerasimova A, Bork P, Kondrashov AS, Sunyaev SR (2010). A method and server for predicting damaging missense mutations. Nat Methods.

[14] Capriotti E, Fariselli P, Casadio R (2005). I-Mutant2.0: Predicting stability changes upon mutation from the protein sequence or structure. Nucleic Acids Res.

[15] Wang XH, Wang HM, Zhao BL, Yu P, Fan ZC (2014). Rescue of defective MC4R cell-surface expression and signalling by a novel pharmacoperone Ipsen 17. J Mol Endocrinol.

[16] Wang CL, Liang H, Wang HJ, Fu JF, Hebebrand J, Hinney A (2006). Several mutations in the melanocortine 4 receptor gene are associated with obesity in Chinese children and adolescents. J Endocrinol Invest.

[17] Michaud JL, Rosenquist T, May NR, Fan CM (1998). Development of neuroendocrine lineages requires the bHLH-PAS transcription factor SIM1. Genes Dev.

[18] Tolson KP, Gemelli T, Gautron L, Elmquist JK, Zinn AR, Kublaoui BM (2010). Postnatal Sim1 deficiency causes hyperphagic obesity and reduced Mc4R and oxytocin expression. J Neurosci.

[19] Holder JL Jr, Butte NF, Zinn AR (2000). Profound obesity associated with a balanced translocation that disrupts the SIM1 gene. Hum Mol Genet.

[20] Faivre L, Cormier -Daire V, Lapierre JM, Colleaux L, Jaquemont S, Genevieve D, Saunier P, Munnich A, Turleau C, Romana S, Prieur M, De Blois MC, Vekemans M (2002). Deletion of the SIM1 gene (6q16.2) in a patient with Prader-Willi -like phenotype. J Med Genet.

[21] Varela MC, Simoes-Sato AY, Kim CA, Bertola DR, De Castro CI, Koiffmann CP (2006). A new case interstitial 6q16.2 deletion in a patient with PraderWilli like phenotype and investigation of SIM1 gene deletion in 87 patients with syndromic obesity. Eur J Med Genet.

[22] Michaud JL, Boucher JL, Melnyk A, Gauthier F, Goshu E, Lévy E, Mitchell GA, Himms-Hagen J, Fan CM (2001). SIM1 haploinsufficiency causes hyperphagia, obesity and reduction of the paraventricular nucleus of the hypothalamus. Hum Mol Genet.

[23] Zegers D, Becker S, Hendrickx R, Van Camp JK, de Craemer V, Verrijken A, Van Hoorenbeeck K, Verhulst SL, Rooman RP, Desager KN, Massa G, Van Gaal LF, Van Hul W (2014). Mutation screen of the SIM1 gene in pediatric patients with early-onset obesity. Int J Obesity (Lond).

[24] Ramachandrappa S, Raimondo A, Cali AM, Keogh JM, Henning E, Saeed S, Thompson A, Garg S, Bochukova EG, Brage S, Trowse V, Wheeler E, Sullivan AE, Dattani M, Clayton PE, Datta V, Bruning JB, Wareham NJ, O’Rahilly S, Peet DJ, Barroso I, Whitelaw ML, Farooqi IS (2013). Rare variants in single-minded 1(SIM1 are associated with severe obesity. J Clin Invest.

[25] Bonnefond A, Raimondo A, Stutzmann F, Ghoussaini M, Ramachandrappa S, Bersten DC, Durand E, Vatin V, Balkau B, Lantieri O, Raverdy V, Pattou F, Van Hul W, Van Gaal L, Peet DJ, Weill J, Miller JL, Horber F, Goldstone AP, Driscoll DJ, Bruning JB, Meyre D, Whitelaw ML, Froguel P (2013). Loss-of-function mutations in SIM1 contribute to obesity and Prader- Willi- like features. J Clin Invest.

[26] Smith AI, Funder JW (1998). Proopiomelanocortin processing in the pituitary, central nervous system, and peripheral tissues. Endocr Rev.

[27] Castro MG, Morrison E (1997). Post-translational processing of proopiomelanocortin in the pituitary and in the brain. Crit Rev Neurobiol.

[28] Challis BG, Pritchard LE, Creemers JWM, Delplanque J, Keogh JM, Luan J, Wareham NJ, Yeo GS, Bhattacharyya S, Froguel P, White A, Farooqi IS, O’Rahilly S (2002). A missense mutation disrupting a dibasic prohormone processing site in pro-opiomelanocortin (POMC) increases susceptibility to early-onset obesity through a novel molecular mechanism. Hum Mol Genet.

[29] Yaswen L, Diehl N, Brennan MB, Hochgeschwender U (1999). Obesity in the mouse model of proopiomelanocortin deficiency responds to peripheral melanocortin. Nat Med.

[30] Krude H, Biebermann H, Luck W, Horn R, Brabant G, Grüters A (1998). Severe eraly-onset obesity, adrenal insufficiency, and red hair pigmentation caused by POMC mutations in humans. Nat Genet.

[31] Creemers JW, Lee YS, Oliver RL, Bahceci M, Tuzcu A, Gokalp D, Keogh J, Herber S, White A, O’Rahilly S, Farooqi IS (2008). Mutations in the amino-terminal region of proopiomelanocortin (POMC) in patients with early-onset obesity impair POMC sorting to the regulated secretory pathway. J Clin Endocrinol Metab.

[32] Dong W, Seidel B, Marcinkiewicz M, Chretien M, Seidah NG, Day R (1997). Cellular localization of the prohormone convertases in the hypothalamic paraventricular and supraoptic nuclei: selective regulation of PC1 in corticotropin-releasing hormone parvocellular neurons mediated by glucocorticoids. J Neurosci.

[33] Farooqi IS, Volders K, Stanhope R, Heuschkel R, White A, Lank E, Keogh J, O’Rahilly S, Creemers JW (2007). Hyperphagia and eraly onset obesity due to a novel homozygous missense mutation in prohormone convertase 1/3. J Clin Endocrinol Metab.

[34] Philippe J, Stijnen P, Meyre D, De Graeve F, Thuillier D, Delplanque J, Gyapay G, Sand O, Creemers JW, Froguel P, Bonnefond A (2015). A nonsense loss-of-function mutation in PCSK1 contributes to dominantly inherited human obesity. Int J Obes.

[35] Benzinou M, Creemers JW, Choquet H, Lobbens S, Dina C, Durand E, Guerardel A, Boutin P, Jouret B, Heude B, Balkau B, Tichet J, Marre M, Potoczna N, Horber F, Le Stunff C, Czernichow S, Sandbaek A, Lauritzen T, Borch-Johnsen K, Andersen G, Kiess W, Körner A, Kovacs P, Jacobson P, Carlsson LM, Walley AJ, Jørgensen T, Hansen T, Pedersen O, Meyre D, Froguel P (2008). Common nonsynonymous variants in PCSK1 confer risk of obesity. Nature Gene.

[36] Farooqi IS, Keofh JM, Yeo GS, Lank EJ, Cheetham T, O’Rahilly S (2003). Clinical spectrum of obesity and mutations in the melanocortin 4 receptor gene. New Engl J Med.

[37] Vaisse C, Clement K, Durand E, Hercberg S, Guy-Grand B, Frougel P (2000). Melanocortin-4 receptor mutations are frequent and heterogenous cause of morbid obesity. J Clin Invest.

[38] Miraglia Del Giudice E, Cirillo G, Nigro V, Santoro N, D’Urso L, Raimondo P, Cozzolino D, Scafato D, Perrone L (2002). Low frequency of melanocortin-4-receptor (MC4R) mutations in a Mediterranean population with early-onset obesity. Int J Obes Related Metab Disord.

[39] Kobayashi H, Ogawa Y, Shintani M, Ebihara K, Shimodahira M, Iwakura T, Hino M, Ishihara T, Ikekubo K, Kurahachi H, Nakao K (2002). A Novel homozygous missense mutation of melanocortin-4 receptor (MC4R) in a Japanese woman with severe obesity. Diabetes.

[40] Nijenhuis WA, Garner KM, van Rozen RJ, Adan RA (2003). Poor cell surface expression of human melanocortin-4 receptor mutations associated with obesity. J Biol Chem.

[41] Larsen LH, Echwald SM, Sorensen TI, Andersen T, Wulff BS, Pedersen O (2005). Prevelance of mutations and fonctional analyses of melanocortin4 receptor variants identified among 750 men with juvenil -onset obesity. J Clin Endocrinol Metab.

[42] Dubern B, Clement K, Pelloux V, Froguel P, Girardet J, Guy-Grand B, Tounian P (2001). Mutational analsis of melanocortin 4 receptor, agouti-related protein and alfa-melanocyte -stimulating hormone genes in severely obese children. J Pediatr.

[43] Farooqi IS, Wangensteen T, Collins S, Kimber W, Matarese G, Keogh JM, Lank E, Bottomley B, Lopez-Fernandez J, Ferraz-Amaro I, Dattani MT, Ercan O, Myhre AG, Retterstol L, Stanhope R, Edge JA, McKenzie S, Lessan N, Ghodsi M, De Rosa V, Perna F, Fontana S, Barroso I, Undlien DE, O’Rahilly S (2007). Clinical and molecular genetic spectrum of congenital deficiency of the leptin receptor. N Engl J Med.

[44] Gill R, Cheung YH, Shen Y, Lanzano P, Mirza NM, Ten S, Maclaren NK, Motaghedi R, Han JC, Yanovski JA, Leibel RL, Chung WK (2014). Whole-exome sequencing identifies novel LEPR mutations in individuals with severe eraly-onset obesity. Obesity.

[45] Foucan L, Larifla L, Durand E, Rambhojan C, Armand C, Michel CT, Billy R, Dhennin V, De Graeve F, Rabearivelo I, Sand O, Lacorte JM, Froguel P, Bonnefond A (2018). High Prevalence of Rare Monogenic Forms of Obesity in Obese Guadeloupean Afro-Caribbean Children. J Clin Endocrinol Metab.

